# Heightened circulating levels of antimicrobial peptides in tuberculosis—Diabetes co-morbidity and reversal upon treatment

**DOI:** 10.1371/journal.pone.0184753

**Published:** 2017-09-14

**Authors:** Nathella Pavan Kumar, Kadar Moideen, Vijay Viswanathan, Shanmugam Sivakumar, Pradeep A. Menon, Hardy Kornfeld, Subash Babu

**Affiliations:** 1 National Institutes of Health—NIRT—International Center for Excellence in Research, Chennai, India; 2 Prof. M. Viswanathan Diabetes Research Center, Chennai, India; 3 National Institute for Research in Tuberculosis, Chennai, India; 4 University of Massachusetts Medical School, Worcester, Massachusetts, United States of America; 5 LPD, NIAID, NIH, Bethesda, Maryland, United States of America; Bose Institute, INDIA

## Abstract

**Background:**

The association of antimicrobial peptides (AMPs) with tuberculosis—diabetes comorbidity (PTB-DM) is not well understood.

**Methods:**

To study the association of AMPs with PTB-DM, we examined the systemic levels of cathelicidin (LL37), human beta defensin– 2 (HBD2), human neutrophil peptides 1–3, (HNP1-3) and granulysin in individuals with either PTB-DM, PTB, latent TB (LTB) or no TB infection (NTB).

**Results:**

Circulating levels of cathelicidin and HBD2 were significantly higher and granulysin levels were significantly lower in PTB-DM compared to PTB, LTB or NTB, while the levels of HNP1-3 were significantly higher in PTB-DM compared to LTB or NTB individuals. Moreover, the levels of cathelicidin and/or HBD2 were significantly higher in PTB-DM or PTB individuals with bilateral and cavitary disease and also exhibited a significant positive relationship with bacterial burden. Cathelidin, HBD2 and HNP1-3 levels exhibited a positive relationship with HbA1c and/or fasting blood glucose levels. Finally, anti-tuberculosis therapy resulted in significantly diminished levels of cathelicidin, HBD2, granulysin and significantly enhanced levels of HNP1-3 and granulysin in PTB-DM and/or PTB individuals.

**Conclusion:**

Therefore, our data demonstrate that PTB-DM is associated with markedly enhanced levels of AMPs and diminished levels of granulysin.

## Introduction

Pulmonary tuberculosis (PTB), a disease caused by the human pathogen *Mycobacterium tuberculosis*, has recently joined HIV/AIDS as the world’s deadliest infectious disease and affects more than 10 million people worldwide [1]. Type 2 diabetes mellitus (DM) is rapidly emerging as one of the world’s most common non-communicable health disease and afflicts over 415 million people worldwide [1]. The confluence of PTB and DM poses a major public health problem due to the overlap in the geographical distribution of both these diseases, especially in China and India [2,3]. Moreover, DM is known to promote the risk of acquisition of PTB and also adversely affect outcomes, including treatment failure, relapse and death [2,4,5]. Both PTB and PTB-DM now face the additional challenge of emerging drug resistance, including multi-drug resistance and extensive drug resistance [6]. Therefore, newer strategies to combat the problem of PTB and PTB-DM are crucially necessary.

Antimicrobial peptides (AMPs), also called host defense peptides, are innate immune effectors that play a vital role in the host defense mechanism against invading pathogens [7]. They are usually short in length (20–60 amino acid residues), exhibit a cationic and amphipathic nature and have activity against bacteria, fungi and viruses [8]. It has been shown that AMPs have high anti-mycobacterial activity but low immunogenicity and are therefore promising therapeutic agents [9,10]. Few studies have examined the effect of treatment on the systemic levels of AMPs in PTB or PTB-DM. Among the AMP family, four of the most prominent are cathelicidin (LL37), human beta defensing– 2 (HBD2), human neutrophil peptide 1–3 (HNP1-3) and granulysin [7]. However, a detailed examination of the association of AMPs with PTB or PTB-DM and their relationship to disease pathology or bacterial burdens has not been performed. We therefore postulated that one potential mechanism for increased susceptibility of DM individuals to TB could be a systemic diminution in the levels of AMPs in PTB-DM individuals.

We performed a detailed examination of the association of the systemic levels of cathelicidin, HDB2, HNP1-3 and granulysin in PTB-DM and PTB individuals and compared them to those with latent TB (LTB) or no TB (NTB). In addition, we examined the AMP levels in those with no TB but DM alone (DM). We demonstrate heightened levels of cathelicidin, HBD2 and HNP1-3 and diminished levels of granulysin in PTB-DM and PTB. We also demonstrate an association of cathelicidin and HBD2 with the extent and severity of lung disease and with bacterial burdens, as well as correlations of AMPs with glycemic parameters. Finally, we show a trend towards normalization in the systemic concentrations of these AMPs with standard anti-tuberculosis treatment in both PTB-DM and PTB individuals.

## Materials and methods

### Ethics statement

This study was approved by the Ethics Committees of the Prof. M. Viswanathan Diabetes Research Center and the National Institute for Research in Tuberculosis (NIRT). Written informed consent was obtained from all participants.

### Study population

Plasma samples were collected from 44 individuals with active pulmonary TB and diabetes mellitus (PTB-DM), 44 individuals with active pulmonary TB and no diabetes (PTB), 30 individuals with latent TB (LTB), 30 individuals with no TB or diabetes (NTB) and 30 individuals with diabetes mellitus (DM), recruited in Chennai, India. The diagnosis of PTB was based on smear and culture positivity for *M*. *tuberculosis*. Chest X-rays were used to define cavitary disease as well as unilateral vs bilateral involvement at enrollment. Smear grades were used to estimate bacterial burden and classified as 1+, 2+ and 3+. At the time of enrollment, all active TB cases had no record of prior TB disease or prior anti-TB treatment (ATT). DM was diagnosed on the basis of oral glucose tolerance test and/or glycated hemoglobin (HbA1c) levels (for known diabetics), according to World Health Organization criteria. LTB diagnosis was based on tuberculin skin test (TST) and Quantiferon TB-Gold in Tube ELISA positivity, absence of chest radiograph abnormalities or pulmonary symptoms and negative sputum smear. A positive TST result was defined as an induration of at least 12mm in diameter to minimize false positivity due to exposure to environmental mycobacteria. NTB individuals were asymptomatic with normal chest X-rays, negative TST (induration < 5 mm in diameter) and Quantiferon ELISA results. No NTB individuals exhibited signs or symptoms of any associated lung or systemic disease. All individuals were BCG vaccinated, HIV negative had normal body mass index. The study groups were similar with regard to age and gender; the baseline characteristics of the study participants are shown in Table 1. Standard ATT was administered to PTB-DM and PTB individuals using the directly observed treatment, short course (DOTS) strategy. At 6 months following ATT initiation, fresh plasma samples were obtained. All PTB-DM and PTB individuals were culture negative at the end of ATT.

**Table 1:** Demographics of the study groups and biochemical parameters in TB-DM and TB.

Study Demographics	PTB-DM	PTB	LTB	NTB	DM
**No. of subjects recruited**	44	44	30	30	30
**Gender (Male / Female)**	33/11	37/7	18/12	16/14	20/10
**Median Age (Range)**	50 (34–70)	41 (25–67)	43 (18–65)	36 (18–65)	47 (18–63)
**Smear Grade: 0/1+/2+/3+**	0/16/14/14	0/18/16/10	NA	NA	NA
**Culture Results: Negative:0/1+/2+/3+**	0/12/17/15	0/28/12/4	NA	NA	NA
**Tuberculin Skin Test (TST)**	Not Done	Not Done	>12mm	<12mm	Not Done
**Interferon gamma release assay**	Not Done	Not Done	Positive	Negative	Not Done
**Random Blood Glucose, mg/dL**	220 (180–448)*	119 (76–137)	98 (78–113)	96 (60–120)	177 (161–401)
**Glycated hemoglobin level, %**	10.2 (6.6–15.6)*	5.6 (5.0–5.8)	5.8 (5.01–6.05)	5.7 (4.7–6.1)	7.86 (6.8–11.11)
**Serum Triglycerides, mg/dL**	108 (33–178)*	74 (39–142)	55 (42–150)	58 (45–144)	99 (41–180)
**Total Cholesterol, mg/dL**	172 (92–258)*	151 (86–192)	135 (80–198)	131 (78–200)	165 (111–285)
**HDL Cholesterol, mg/dL**	37 (20–83)	36 (19–69)	30 (19–75)	32 (21–78)	35 (22–78)
**LDL Cholesterol, mg/dL**	94 (51–165)	83 (49–107)	88 (45–103)	79 (41–98)	98 (55–174)

The values represent the geometric mean (and the range) except for age where the median (and the range) are depicted.

*Represents values that are significantly different in PTB-DM compared to PTB, LTB and NTB.

### ELISA

Circulating levels of HBD2 and HNP 1–3 were measured using Mybiosource ELISA kits. Cathelicidin (LL-37) was measured using Hycult biotech and Granulysin was measured using Duoset ELISA Development System (R&D Systems) in plasma samples. The lowest detection limits were as follows: HBD2, 15.6 pg/mL; HNP1-3, 0.625 ng/mL; LL-37, 0.1 ng/mL, Granulysin, 15.625 pg/mL

### Statistical analysis

Geometric means (GM) were used for measurements of central tendency. Statistically significant differences between the three groups were analyzed using the Kruskal-Wallis test with Dunn’s correction for multiple comparisons. The Mann-Whitney test was used to compare antimicrobial peptides concentrations between the individuals with pulmonary TB with unilateral or bilateral lung lesions and cavitary or non-cavitary disease. Linear trend post-test was used to compare antimicrobial peptides concentrations with sputum smear grades (reflecting bacterial burdens). Wilcoxon signed rank test was used to compare antimicrobial peptides concentrations before and after ATT and Holm’s correction for multiple comparisons was applied. Analyses were performed using Graph-Pad PRISM Version 5.01.

## Results

### Study population characteristics

The baseline characteristics including demographic and biochemical features of the study population are shown in Table 1. As can be seen, compared to PTB, those with PTB-DM had significantly higher levels of random glucose, glycated hemoglobin, serum cholesterol and triglycerides. No significant differences were observed in age, sex, BMI, smear or culture grades at baseline between the 2 groups. The demographic and biochemical features of the other groups in the study, including LTB, NTB and DM are also shown in Table 1.

### Heightened circulating levels of cathelicidin, HBD2, HNP1-3 and diminished levels of granulysin in PTB-DM

**Fig 1:**
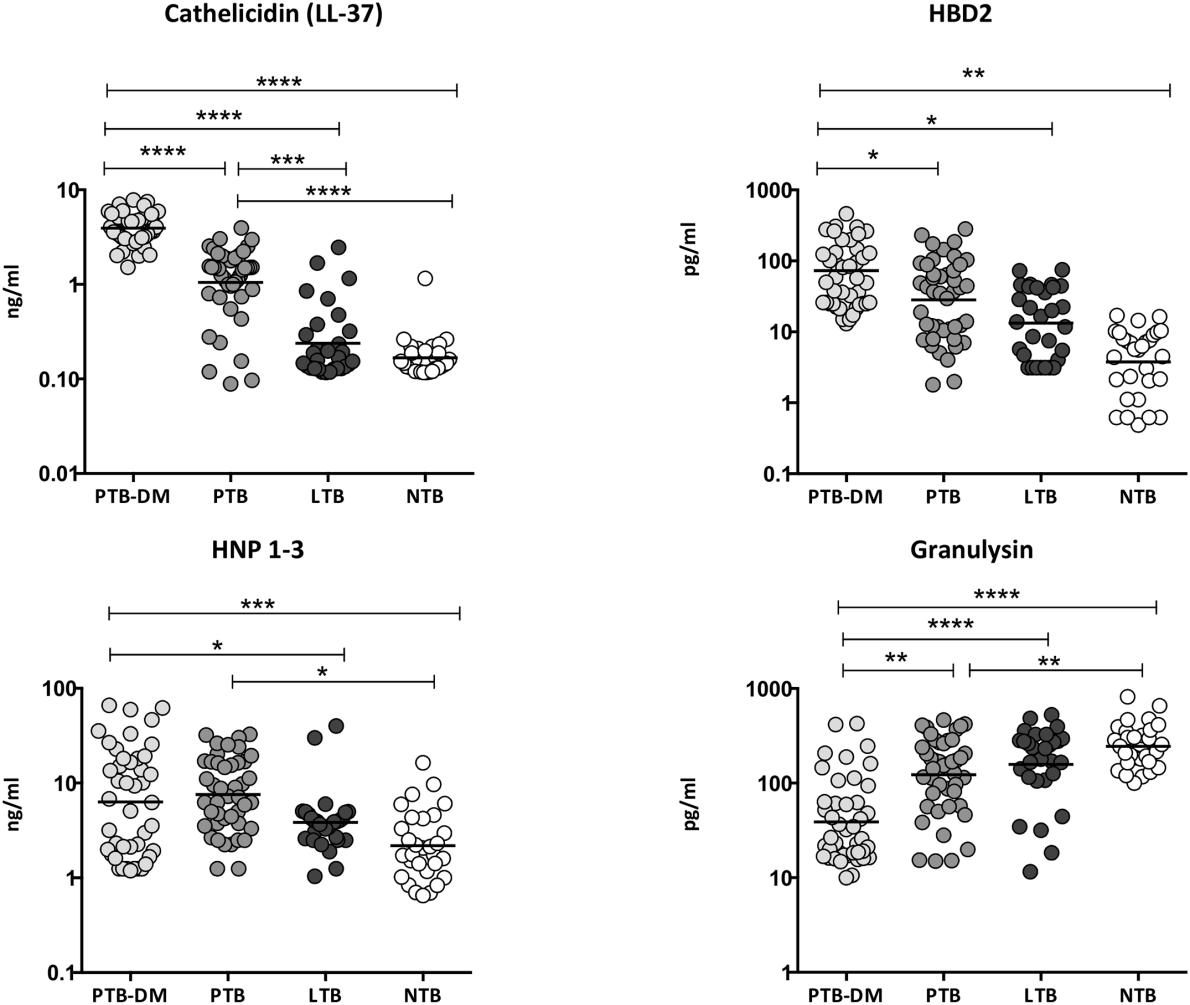
Elevated circulating levels of AMPs in PTB-DM and PTB individuals. The plasma levels of cathelicidin (LL37), HBD2, HNP1-3 and granulysin were measured in PTB-DM (n = 44), PTB (n = 44), LTB (n = 30) and NTB (n = 30) individuals. The data are presented as scatter plots with each circle representing a single individual. P values were calculated using the Kruskal—Wallis test with Dunn’s post—hoc for multiple comparisons.

To determine the systemic levels of circulating AMPs in PTB-DM and PTB, we measured the circulating levels of cathelicidin, HBD2, HNP1-3 and granulysin in PTB-DM, PTB, LTB and NTB individuals (Fig 1). As shown, the circulating levels of cathelicidin (Geometric Mean of 3.9 ng/ml in PTB-DM vs 1.1 ng/ml in PTB, 0.23 ng/ml in LTB and 0.17 ng/ml in NTB), HBD2 (GM of 72.6 pg/ml in PTB-DM vs 28.2 pg/ml in PTB, 13.3 pg/ml in LTB and 3.8 pg/ml in NTB) and HNP1-3 (GM of 6.3 ng/ml in PTB-DM vs 3.8 ng/ml in LTB and 2.2 ng/ml in NTB) were significantly higher in PTB-DM compared to PTB and/or LTB and NTB individuals. In contrast, the circulating levels of granulysin (GM of 38.8 pg/ml in PTB-DM vs 122.9 pg/ml in PTB, 158.3 pg/ml in LTB and 245.1 pg/ml in NTB) was significantly lower in PTB-DM compared to PTB, LTB and NTB individuals. Similarly, the circulating levels of cathelicidin and HNP1-3 were significantly higher and those of granulysin significantly lower in PTB compared to NTB individuals. Thus, PTB-DM is associated with elevated systemic levels of circulating AMPs, with the exception of granulysin.

### Enhanced circulating levels of cathelicidin and HBD2 in PTB-DM compared to DM

To compare the systemic levels of circulating AMPs between PTB-DM and DM, we measured the circulating levels of cathelicidin, HBD2, HNP1-3 and granulysin in PTB-DM and DM alone individuals (Table 2). As shown, the circulating levels of cathelicidin (GM of 3.9 ng/ml in PTB-DM vs 1.2 ng/ml in DM) and HBD2 (GM of 72.6 pg/ml in PTB-DM vs 5.2 pg/ml in DM) were significantly higher in PTB-DM compared to DM individuals. Thus, PTB-DM associated enhanced levels of AMPs are mainly related to TB disease.

**Table 2:** Circulating levels of AMPs in PTB-DM and DM individuals.

	PTB-DM	DM	pValue
**LL-37**	3.94 (1.51–7.82)	1.21 (0.11–4.06)	**p<0.0001**
**HBD2**	72.63 (13.13–463.01)	5.21 (0.42–109.53)	**p<0.0001**
**HNP1-3**	2.14 (0.65–16.39)	1.98 (0.25–18.59)	p = 0.3487
**Granulysin**	244.04 (100–818.83)	153.53 (8.12–688.43)	p = 0.1469

The values represent thte geometric mean (and the range).

### Circulating AMPs are markers of disease severity and bacterial burdens in PTB-DM

**Fig 2:**
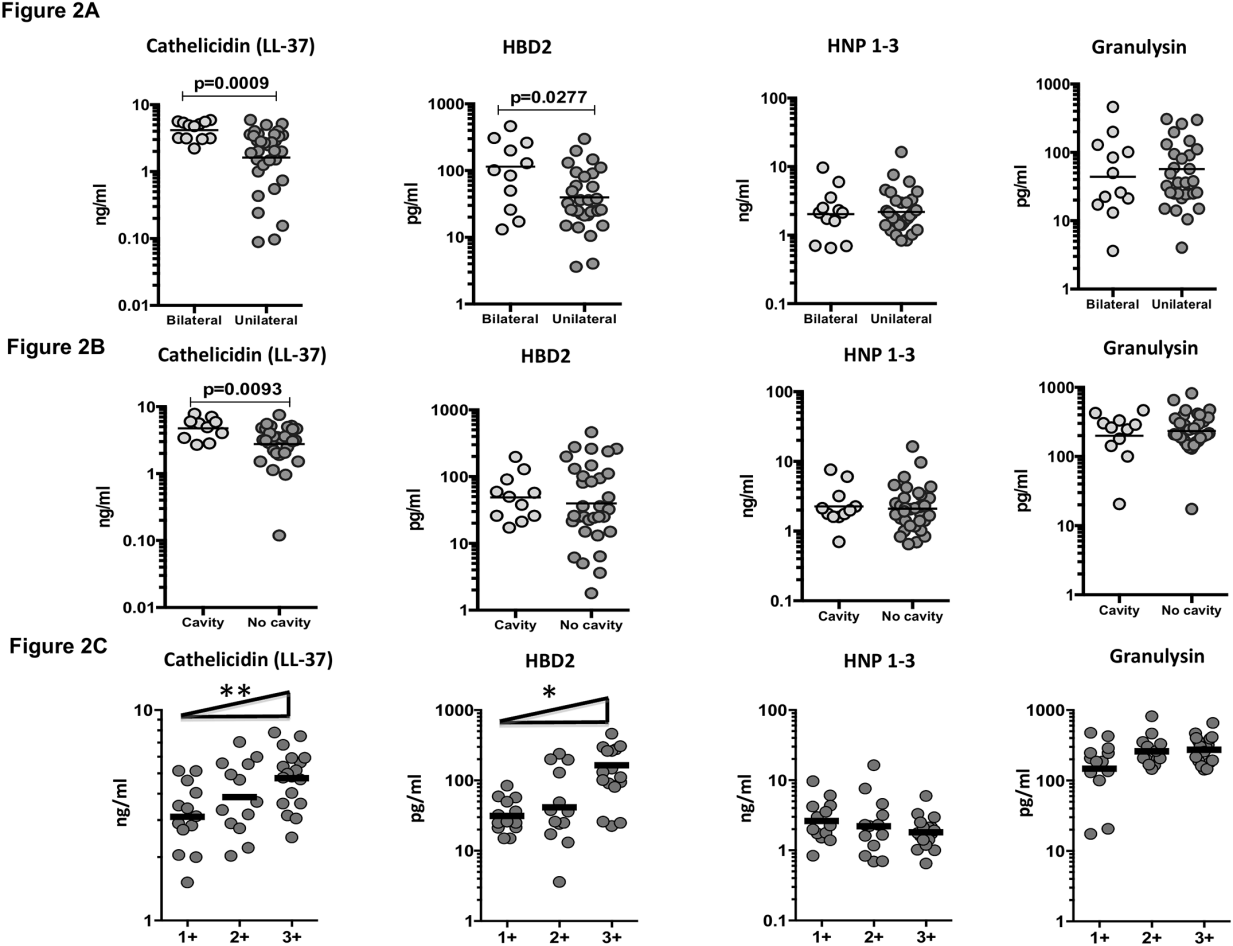
Elevated circulating levels of cathelicidin and HBD2 in bilateral and cavitary disease in PTB-DM individuals and relationship to bacterial burden. (A) The plasma levels of cathelicidin (LL37), HBD2, HNP1-3 and granulysin were measured in PTB-DM individuals with bilateral vs unilateral disease. (B) The plasma levels of cathelicidin (LL37), HBD2, HNP1-3 and granulysin were measured in PTB-DM individuals with cavitary vs non-cavitary disease. (C) The relationship between the plasma levels of cathelicidin (LL37), HBD2, HNP1-3 and granulysin and smear grades as estimated by sputum smears was examined in PTB-DM individuals. The data are presented as scatter plots with each circle representing a single individual. P values were calculated using the Mann-Whitney test or the Linear trend post—test.

To determine the association between the systemic levels of circulating AMPs and disease severity in PTB-DM, we measured the circulating levels of cathelicidin, HBD2, HNP1-3 and granulysin in PTB-DM individuals with unilateral vs bilateral disease and cavitary vs non-cavitary disease. As shown in Fig 2A, the circulating levels of cathelicidin (GM of 4.1 ng/ml in bilateral vs. 1.6 ng/ml in unilateral disease) and HBD2 (GM of 114.2 pg/ml in bilateral vs. 39.8 pg/ml in unilateral disease) were significantly higher in PTB-DM individuals with bilateral disease compared to those with unilateral disease. Similarly, as shown in Fig 2B, the circulating levels of cathelicidin (GM of 4.7 ng/ml in cavitary vs. 2.7 ng/ml in non-cavitary disease) were significantly higher in PTB-DM individuals with cavitary disease compared to those without.

To determine the association of circulating AMPs and bacterial burdens, we performed a correlation of the circulating levels of cathelicidin, HBD2, HNP1-3 and granulysin in PTB-DM individuals with smear grades. As shown in Fig 2C, both cathelicidin and HBD2 exhibited a significant positive correlation with smear grades in PTB-DM individuals, indicating a positive association of these factors with bacterial burdens. Thus, both disease severity and bacterial burden in PTB-DM is associated with elevated systemic levels of circulating AMPs.

### Circulating AMPs are also markers of disease severity and bacterial burden in PTB

**Fig 3:**
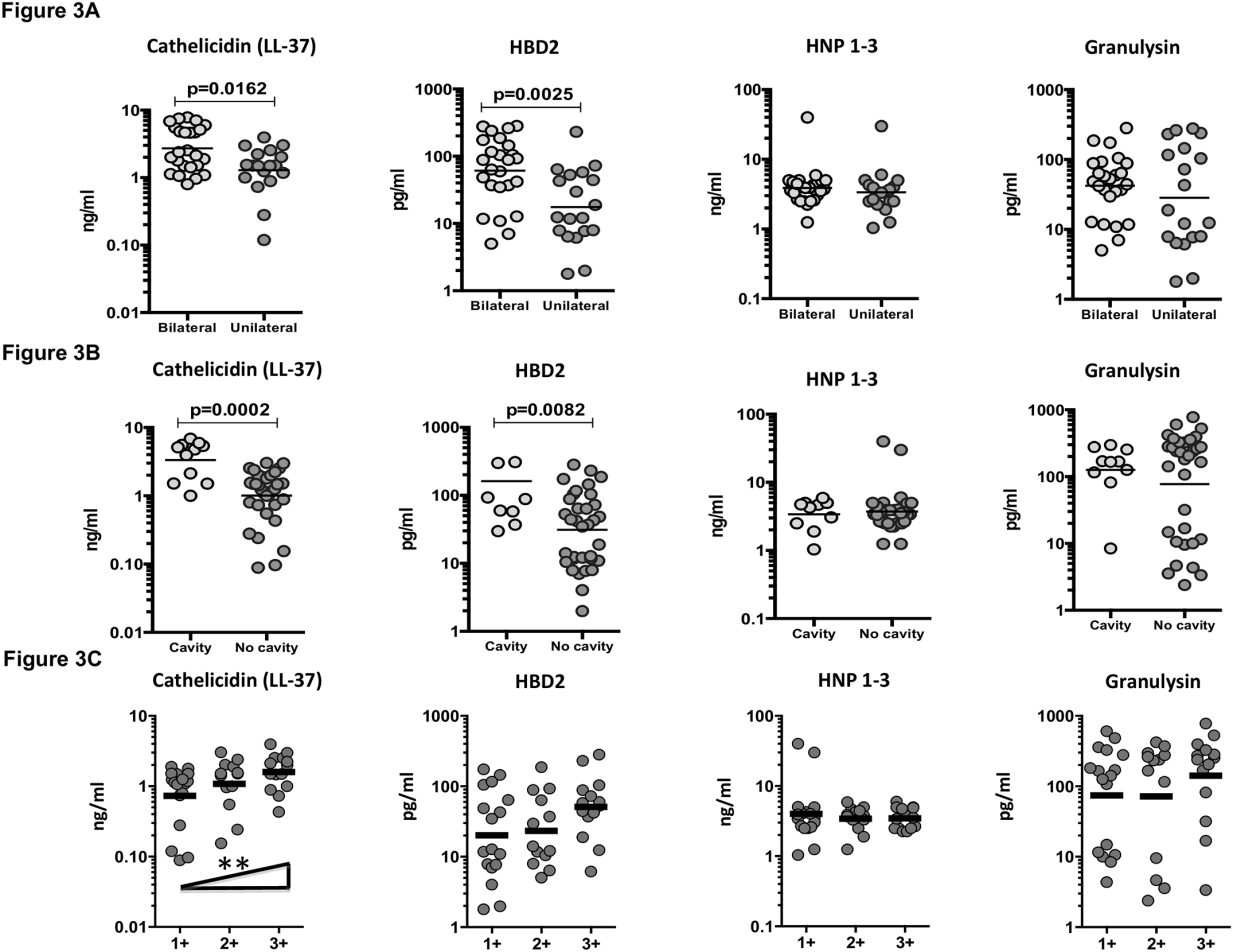
Elevated circulating levels of cathelicidin and HBD2 in bilateral and cavitary disease in PTB individuals and relationship to bacterial burdens. (A) The plasma levels of cathelicidin (LL37), HBD2, HNP1-3 and granulysin were measured in PTB individuals with bilateral vs unilateral disease. (B) The plasma levels of cathelicidin (LL37), HBD2, HNP1-3 and granulysin were measured in PTB individuals with cavitary vs non-cavitary disease. (C) The relationship between the plasma levels of cathelicidin (LL37), HBD2, HNP1-3 and granulysin and smear grades as estimated by sputum smears was examined in PTB individuals. The data are presented as scatter plots with each circle representing a single individual. P values were calculated using the Mann-Whitney test or the Linear trend post—test.

To determine the association between the systemic levels of circulating AMPs and disease severity in PTB without DM, we measured the circulating levels of cathelicidin, HBD2, HNP1-3 and granulysin in PTB individuals with unilateral vs bilateral disease and cavitary vs. non-cavitary disease. As shown in Fig 3A, the circulating levels of cathelicidin (GM of 2.7 ng/ml in bilateral vs. 1.3 ng/ml in unilateral disease) and HBD2 (GM of 61.2 pg/ml in bilateral vs. 17.5 pg/ml in unilateral disease) were significantly higher in PTB individuals with bilateral disease compared to those with unilateral disease. Similarly, as shown in Fig 3B, the circulating levels of cathelicidin (GM of 3.3 ng/ml in cavitary vs. 1.0 ng/ml in non-cavitary disease) and HBD2 (GM of 161.6 pg/ml in cavitary vs. 31.2 pg/ml in non-cavitary disease) were significantly higher in PTB individuals with cavitary disease compared to those without.

To determine the association of circulating AMPs and bacterial burdens, we performed a correlation of the circulating levels of cathelicidin, HBD2, HNP1-3 and granulysin in PTB individuals with smear grades. As shown in Fig 3C, cathelicidin (but not the other AMPs) exhibited a significant positive correlation with smear grades in PTB individuals, indicating a positive association with bacterial burdens. Thus, both disease severity and bacterial burden in PTB is associated with elevated systemic levels of circulating AMPs.

### Circulating cathelicidin and HBD2 exhibit a positive relationship while granulysin exhibits a negative relationship with HbA1c or fasting blood glucose levels in PTB

**Fig 4:**
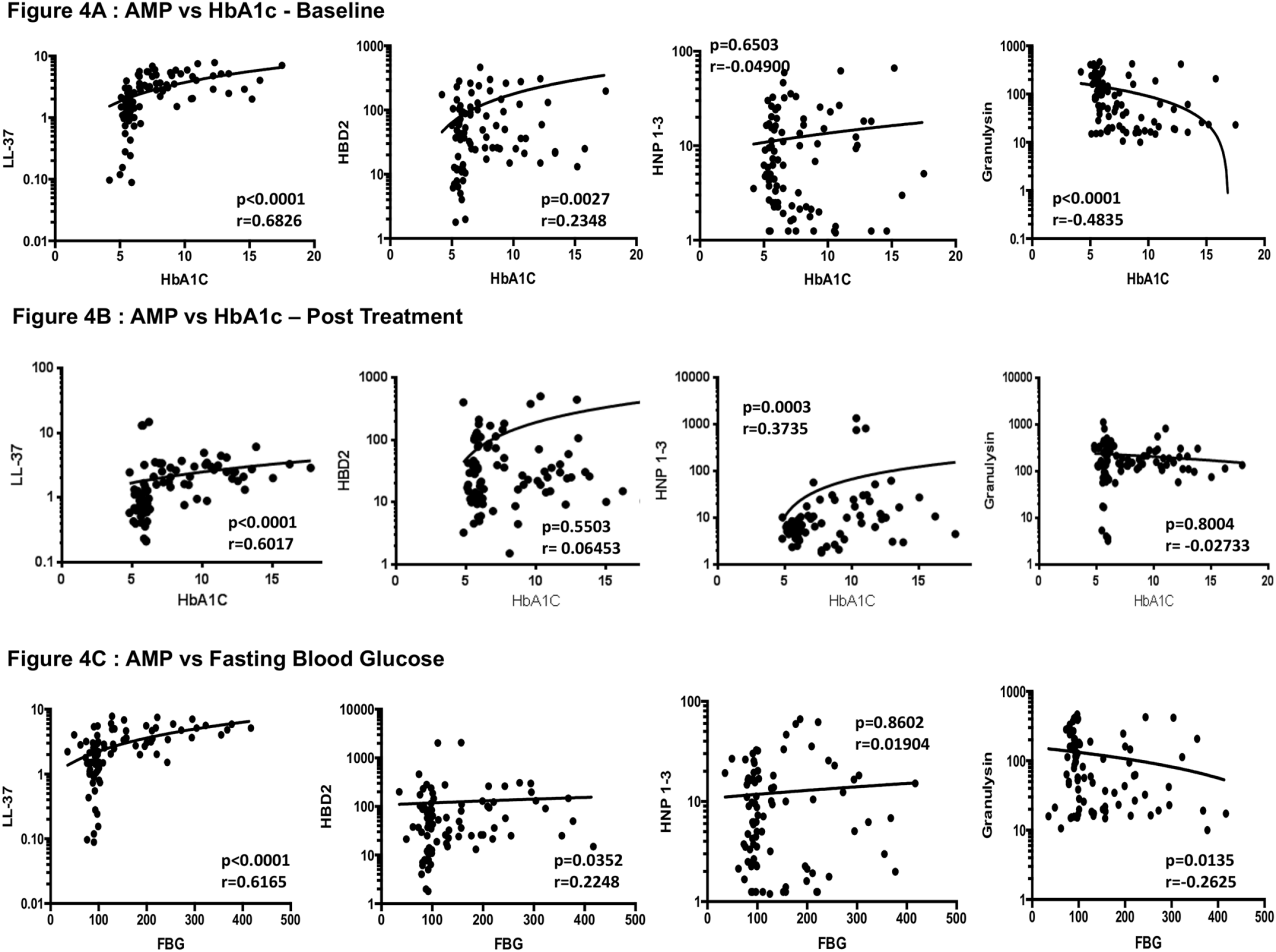
Significant correlation between circulating levels of AMPs and glycemic parameters in all PTB individuals. (A) The relationship between the plasma levels of cathelicidin (LL37), HBD2, HNP1-3 and granulysin and HbA1c levels was examined in all PTB individuals at baseline (B) The relationship between the plasma levels of cathelicidin (LL37), HBD2, HNP1-3 and granulysin and HbA1c levels was examined in all PTB individuals at the end of anti-TB treatment. (C) The relationship between the plasma levels of cathelicidin (LL37), HBD2, HNP1-3 and granulysin and fasting blood glucose (FBG) levels was examined in all PTB individuals at baseline. The data are presented as scatter plots with each circle representing a single individual. P values were calculated using the Spearman Rank Correlation.

To determine the association between systemic levels of circulating AMPs and glycemic control in PTB-DM and PTB, we examined the relationship between the circulating levels of cathelicidin, HBD2, HNP1-3 and granulysin in all PTB individuals with HbA1c levels (Fig 4A). As shown, the systemic levels of cathelicidin and HBD2 exhibited a significant positive relationship, while granulysin exhibited a significantly negative relationship with HbA1c levels in PTB individuals, indicating a significant association of these factors with poor glycemic control. Interestingly, even at the end of anti-TB treatment, the systemic levels of cathelicidin and HBD2 (and in addition, HNP1-3) exhibited a significantly positive relationship with HbA1c levels in PTB (Fig 4B). To determine the association between systemic levels of circulating AMPs and glycemic control in PTB, we also examined the relationship between the circulating levels of cathelicidin, HBD2, HNP1-3 and granulysin in all PTB individuals with fasting blood glucose levels (Fig 4C). As shown, the systemic levels of cathelicidin and HBD2 exhibited a significant positive relationship, while granulysin exhibited a significantly negative relationship with fasting blood glucose levels in PTB individuals, also indicating a significant association of these factors with poor glycemic control.

### Anti-tuberculosis treatment results in normalizes circulating levels of AMPs in PTB-DM and PTB

**Fig 5:**
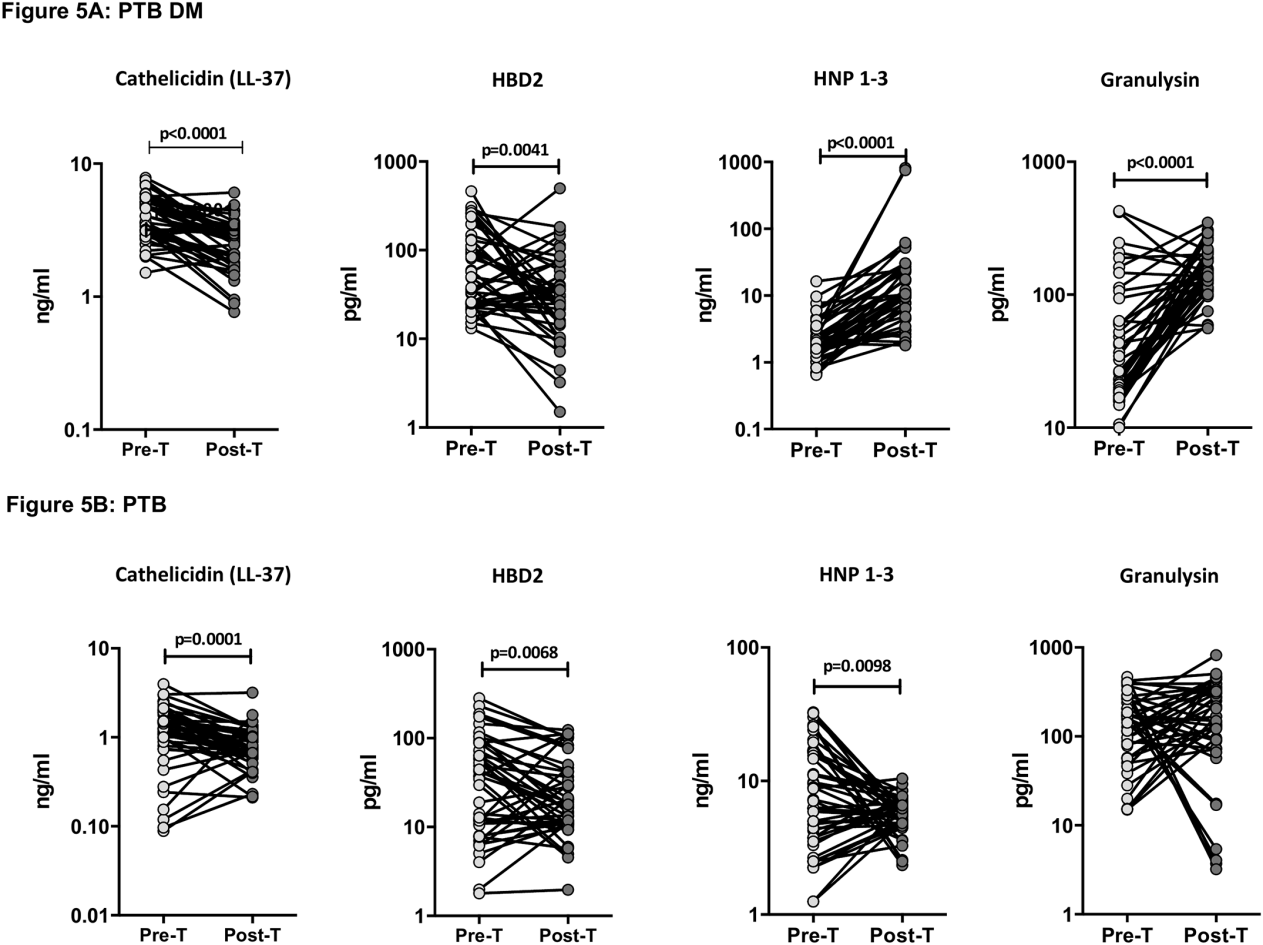
Altered circulating levels of AMPs at the end of standard anti-tuberculosis therapy in PTB-DM and PTB individuals. (A) The plasma levels of cathelicidin (LL37), HBD2, HNP1-3 and granulysin were measured in PTB-DM individuals at baseline (pre-T) and at 6 months of ATT (post-T). (B) The plasma levels of cathelicidin (LL37), HBD2, HNP1-3 and granulysin were measured in PTB individuals at baseline (pre-T) and at 6 months of ATT (post-T). The data are presented as line graphs with each line representing a single individual. P values were calculated using the Wilcoxon signed rank test.

To determine whether the elevated levels of circulating AMPs are directly associated with TB disease, we determined the levels of these factors in PTB-DM and PTB individuals at baseline (pre-T) and at the end of 6 months of anti-tuberculosis treatment (post-T). As shown in Fig 5A, at the end of treatment, the circulating levels of cathelicidin (GM of 2.4 ng/ml at post-T compared to 3.9 ng/ml at pre-T) and HBD2 (GM of 28.5 pg/ml at post-T compared to 72.6 pg/ml at pre-T) were significantly diminished, while the circulating levels of HNP1-3 (GM of 2.4 ng/ml at post-T compared to 3.9 ng/ml at pre-T) and granulysin (GM of 28.5 pg/ml at post-T compared to 72.6 pg/ml at pre-T) were significantly increased compared to pre-treatment levels in PTB-DM individuals. Similarly, as shown in Fig 5B, the circulating levels of cathelicidin (GM of 0.74 ng/ml at post-T compared to 1.1 ng/ml at pre-T) and HBD2 (GM of 20.9 pg/ml at post-T compared to 28.2 pg/ml at pre-T) were significantly decreased, while the levels of HNP1-3 (GM of 5.4 ng/ml at post-T compared to 7.5 ng/ml at pre-T) were significantly increased at post-treatment compared to pre-treatment levels in PTB individuals. Thus, successful treatment of tuberculosis results in a trend towards normalization of circulating AMPs in PTB-DM and PTB.

## Discussion

AMPs are an important component of the innate immunity to pathogens and are expressed mainly in phagocytic cells of the immune system, where they exert microbicidal activity against engulfed pathogens [11]. Many AMPs kill pathogens by disrupting the physical integrity the microbial membrane and/or by translocating across the membrane into the cytoplasm of bacteria to act on intracellular targets [9]. In addition to a direct antimicrobial effect, AMPs can regulate the immune response by a variety of mechanisms including chemotaxis, activation of antigen presenting cells and cytokine induction [12,13]. Recent work has highlighted the importance of AMPs in host defense against TB [14]. In fact, AMPs have been proposed as an attractive therapeutic candidate for anti-mycobacterial therapy [10]. However, the role of AMPs in TB-DM comorbidity has been little studied and hence, we sought to examine their role in this setting.

Cathelicidin is known to elicit a wide range of responses, including both pro- and anti-inflammatory responses, chemoattractant activity, anti-infective activity in the form of induced expression of chemokines, pro-angiogenic activity and pro-apoptotic activity [7]. Cathelicidin is considered to be a key molecule for the control of TB. In vitro studies have clearly demonstrated a pivotal role for cathelicidin in the antimicrobial activity of human macrophages against *M*. *tuberculosis* [15]. Thus, in the presence of vitamin D and TLR2 stimulation, macrophages upregulate the expression of cathelicidin, which then exerts anti-mycobacterial activity [16,17]. Also, vitamin D3 has been shown to induce autophagy in human monocytes/macrophages via the induction of cathelicidin [18]. In addition, cathelicidin expression in alveolar macrophages has also been demonstrated in TB infection [19]. Finally, the levels of cathelicidin has been to be elevated in the in the serum or bronchoalveolar lavage fluid of active TB [20,21].

In agreement with these reports, our study revealed that PTB individuals exhibited significantly higher systemic levels of cathelicidin compared to NTB individuals. In addition, our data also revealed a novel association of cathelicidin levels with the severity of TB disease (as estimated by the bilateral and cavitary disease) and with estimated bacterial burden. Finally, our data revealed that systemic levels of cathelicidin are significantly diminished in PTB individuals following successful ATT. Of additional interest was the find that cathelicidin levels were significantly elevated in PTB-DM individuals compared to PTB, LTB, NTB and DM alone individuals; that these levels were positively correlated with severity of disease and increasing bacterial burdens; that these levels also positively correlated with HbA1c and random blood glucose (indicating an association with poor glycemic control) and that these enhanced levels were significantly diminished following anti—TB treatment. These findings are in line with a previous report on cathelicidin levels being enhanced in PTB-DM individuals [22], but differ from a study that showed decreased CAMP gene expression in these individuals [23].

HBD2 is a human β defensin, which is expressed in epithelial cells and produced by monocytes, macrophages and dendritic cells [24]. The expression of HBD2 is typically induced by pro-inflammatory cytokines via NF-κB activation [25]. HBD2 is known to have pro-inflammatory effects with the ability to cause upregulation of chemokines, CXCL8 and CCL2 [26]. While HBD2 expression is induced in different cell types by mycobacterial infection [27,28], very little is known about the role of HBD2 in PTB or PTB-DM. Our data reveal that HBD2 levels are present at significantly enhanced levels in PTB-DM compared to the other groups, correlates with severity of disease and/or bacterial burdens in PTB and PTB-DM, exhibits a positive relationship with HbA1c levels and is significantly diminished following TB treatment in both PTB and PTB-DM. Thus, HBD2 appears to be associated with pathology and bacterial burdens in PTB-DM and could serve as an important biomarker for both severity of disease and therapeutic responses following treatment.

HNP1-3 are defensins, produced by neutrophils, monocytes, lymphocytes and natural killer cells [24]. They were shown to be capable of killing *M*. *tuberculosis* in vitro [29] and HNP1-3 levels were found to be elevated in plasma and BAL of active TB patients [30]. Our data reveals that HNP1-3 levels are present at significantly enhanced levels in PTB-DM and PTB compared to the other groups, do not correlate with severity of disease and/or bacterial burdens, do not exhibit a relationship with HbA1c levels and are significantly elevated following TB treatment in PTB-DM and PTB individuals. Thus, HNP1-3 levels appear to be associated with active PTB with or without DM, although not directly associated with either severity of disease or bacterial burden. The differences in the association of HBD2 and HNP1-3 with bacterial burdens might be related to the source of these anti-microbial peptides or their site of production.

Granulysin is a protein found in granules of CD8+ T cells and exhibits antimicrobial activity against *M*. *tuberculosis* in vivo and in vitro [31]. It was first shown to kill extracellular *M*. *tuberculosis* directly by altering the membrane integrity and to decrease the viability of intracellular bacteria in combination with perforin [31]. It was later shown that granulysin levels are diminished in adult and pediatric TB, a modulation that is reversible with ATT [32,33]. Our data recapitulate those results and show that circulating granulysin levels were indeed lower in PTB-DM and PTB individuals and that these levels could be enhanced (at least in PTB-DM) by ATT. Thus, among the AMPs examined in this study, granulysin was the one factor that was observed to present at diminished levels in active TB. In addition, granulysin levels exhibited a negative correlation with HBA1c and fasting blood glucose, indicating that granulysin levels were also influenced by glycemic parameters in PTB-DM.

Our data on AMPs overall suggest that modulation and upregulation of AMPs is a typical characteristic of TB-DM co-morbidity. Our data also add to the growing list of evidence indicating heightened immune activation in this immune-metabolic nexus. It is therefore important to study in the expression of AMPs in primary cells under conditions mimicking DM, PTB and PTB-DM in the future. AMPs appear to act as reliable and reproducible biomarkers for therapeutic monitoring of TB-DM disease. Finally, our study reinforces the suggestion that TB-DM co-morbidity is characterized by systemic immune activation, mostly driven by poorly controlled hyperglycemia.

## Supporting information

S1 File Supplementary Data: The raw data for all the figures in the study are provided in the supplementary dataset as a an excel file. (XLSX)
